# Designing National Forest Inventories for Accurate Estimation of Soil Carbon Change

**DOI:** 10.1111/gcb.70868

**Published:** 2026-04-27

**Authors:** Robert W. Buchkowski, Alexander Polussa, Mark A. Bradford

**Affiliations:** ^1^ Department of Biology Western University London Ontario Canada; ^2^ The Forest School, Yale School of the Environment Yale University New Haven Connecticut USA; ^3^ Yale Center for Natural Carbon Capture Yale University New Haven Connecticut USA

**Keywords:** carbon stock variability, ecozone, forest soil carbon change, minimum detectable difference (MDD), National Forest Inventory (NFI), regression to the mean, sampling design, soil carbon monitoring

## Abstract

Detecting changes in forest soil carbon stocks is critical for compiling national carbon budgets, yet remains challenging due to high spatial variability and relatively small temporal changes. Here, we use data from Canada's National Forest Inventory (NFI), which includes repeated measurements of organic and mineral soil horizons across 532 plots. We quantified within‐ and between‐plot variability in soil carbon properties, assessed minimum detectable differences (MDD), and explored design improvements through simulations. Spatial variation in soil carbon stocks was substantial: coefficients of variation were ~40% for mineral and ~70% for organic horizons, with within‐plot comparable to between‐plot variability. Consequently, MDDs were also high, at ~4.1 and 4.6 Mg ha^−1^ 10 year^−1^ for the surface mineral and organic horizons, respectively. This implies that only large, widespread changes would be detectable with the current data. Simulations showed that increasing the number of remeasurement plots to ~700 with four subsamples per plot could reduce MDD to be on par with the current estimate of soil carbon change. Grouping plots by ecozone provided inconsistent benefits at the national level because of ecozones with high spatial heterogeneity. The data also had patterns consistent with the statistical phenomenon of regression to the mean, which implies that any change in carbon stock may be a statistical artifact. Indeed, soil carbon stocks appeared to grow by 2.3 Mg C ha^−1^ 10 year^−1^ during the first remeasurement interval, while a small number of second remeasurement interval data showed a completely unrelated pattern supporting the inference that this first interval change was a statistical artifact. Overall, our analysis of the NFI data suggests that its design characteristics of sampling multiple microplots per main plot and collecting longitudinal data per microplot are critical to providing robust estimates of soil carbon stock changes that can be used in national greenhouse gas inventories.

## Graphical abstract

We use data from Canada's National Forest Inventory (NFI), which includes repeated measurements of organic and mineral soil horizons across 532 plots. We quantified within‐ and between‐plot variability in soil carbon properties, assessed minimum detectable differences (MDD), and explored design improvements through simulations. Our analysis of the NFI data suggests that its design characteristics of sampling multiple microplots per main plot and collecting longitudinal data per microplot are critical to providing robust estimates of soil carbon stock changes.

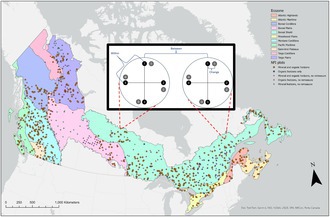

## 1 Introduction

Carbon stocks in forest soils are large, accounting for ~40% of standing forest stocks (Sothe et al. 2022). Changes in these stocks can be consequential for net ecosystem carbon storage (Hararuk et al. 2017; Sothe et al. 2022). Tracking changes in forest soil carbon stocks is therefore necessary for informing fluxes in global carbon cycle models, national greenhouse gas budgets, and for managing the contribution of land use and land management to carbon emissions and removals (Kurz et al. 2009; Mayer et al. 2020; Sanderman et al. 2017).

Quantifying change in soil carbon stocks is notoriously challenging because stocks are highly variable in size—relative to temporal changes—across meter to regional scales (Bradford et al. 2023; Potash et al. 2025). Yet because soil carbon stocks are so large, even small temporal changes can have meaningful consequences for carbon budgeting, shaping whether systems function as carbon sources or sinks. The quantification challenges raise questions about whether national and multi‐national monitoring efforts can accurately and precisely quantify change in soil carbon stocks through empirical remeasurement of soils (Bellamy et al. 2005; Saby et al. 2008). Of particular interest is knowing the number of samples, and the sampling designs, required to quantify changes in soil carbon across time (i.e., monitoring) and attribute them to causal drivers (Potash et al. 2025; Saby et al. 2008; Schrumpf et al. 2011).

Here, we focus on national‐level monitoring of forest soil carbon stocks. We use data from the Canadian National Forest Inventory (NFI), which has sampled and resampled soil organic and mineral horizons from working forests across Canada at spatial and temporal scales that are somewhat unprecedented for soil monitoring (National Forest Inventory 2008, 2021a, 2021b). Since carbon stocks in the organic and mineral horizons are shaped by different processes and have different turnover times, they represent distinct carbon pools for monitoring (e.g., Buchkowski et al. 2024; Hararuk et al. 2017). We ask what sampling numbers and designs are required for accurately estimating change in forest soil carbon stocks in organic and mineral horizons, where our overarching goal is to query current data to inform future empirical sampling in a way that builds confidence that estimates of stock changes are robustly quantified (Bradford et al. 2023; Saby et al. 2008).

We investigate sampling numbers and designs in the context of statistical and field‐sampling realities that, if not addressed, could lead to erroneous conclusions about both the causes and extent of change in soil carbon stocks (Lang et al. 2025; Lark 2009; Lark et al. 2006). Using the current NFI design and data as a case study, we first ascertain the extent to which forest soil monitoring programs provide reliable estimates of change in soil carbon stocks. We next examine how design and replication decisions may provide greater efficiency, accuracy, and precision in estimated changes. To address these two objectives, we use the common approach focused on detecting a minimum threshold change in soil carbon, typically estimated as minimum detectable difference (de Gruijter et al. 2006), and simulate different sampling designs (Potash et al. 2025).

We begin our analysis by describing the measured variation within versus among sites in soil carbon stocks in the mineral and organic horizons to test expectations that stocks are highly variable at the scale of meters (within sites) as well as among sites (tens to thousands of kilometers). We then evaluate the influence of this variability on statistical phenomena such as regression to the mean, where re‐measurement of spatially variable properties can lead to estimates of large changes in soil carbon where none have occurred (Bradford et al. 2023; Lark et al. 2006; Slessarev et al. 2023). We ask how such phenomena affect the reliability of site‐level and sub‐regional estimates of carbon change, when compared to mean change in soil carbon at the national scale of the NFI. We further evaluate whether non‐random decisions related to sampling, such as the extent to which lower sampling effort in sites that are rocky, waterlogged or dangerous, might systematically bias estimates of mean stock change (National Forest Inventory 2008).

## 2 Methods

### 2.1 Dataset

**FIGURE 1:**
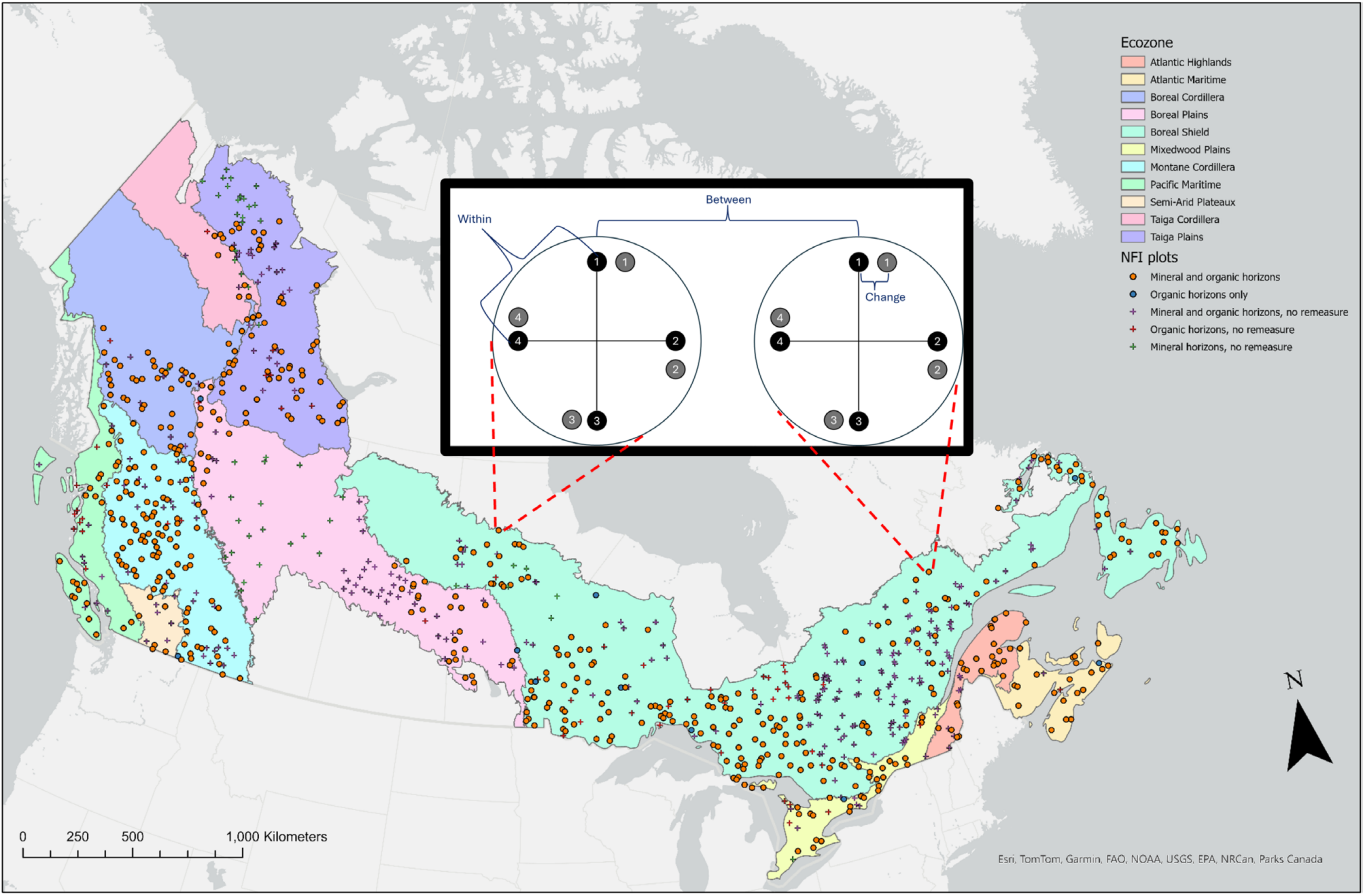
The National Forest Inventory (NFI) plots with soil carbon data. Circular symbols show the plots with at least one soil microplot remeasured, while crosses show those without these data. Colors indicate which horizons have data. Polygons, downloaded from https://ccea‐ccae.org/, show the ecozones in Canada covered by the NFI network plots. The call out box shows the NFI plot sampling design for soil carbon stocks, with brackets marking remeasurement “change,” “between”‐, and “within”‐site standard deviation. NFI plot data are provided by the National Forest Inventory (2021a, 2024).

We explored carbon measurement and re‐measurement data for organic and mineral soil horizons using the Canadian National Forest Inventory (NFI) ground plot data (National Forest Inventory 2021a, 2024). These data are held by the Canadian Forest Service and contain 801 ground plots—one per site—across the country with soil carbon data (Figure 1). Each plot has four microplots. Of these plots, 532 have been remeasured at least once at the same microplot(s), with 428 and 429 having mineral and organic horizon remeasurement, respectively (Figure 1). To facilitate comparisons among our analyses, we included only the 532 plots with at least one remeasurement at one or more microplots. Remeasurements are scheduled to occur every ten years, but remeasurement has occurred as early as 3 years and as late as 19 years since the previous measurement.

NFI samples soil carbon at a plot by taking a sample from up to four microplots, with microplots located at the ends of two 30‐m transects centered at the plot location and running perpendicular to each other (National Forest Inventory 2008). Soil carbon remeasurement occurs at the ends of the same two transects with the microplot location offset by 2‐m clockwise (Figure 1). The offset is necessary to accommodate other microplot sampling that removes understory plant biomass. Soils are sampled in the NFI plots throughout the snow‐free season, May to November, and we analyze all samples because there appeared to be no seasonal bias in SOC stocks (see Figure S1).

Soil organic and mineral carbon are sampled at the same point (National Forest Inventory 2008). Specifically, the organic horizon is sampled as a 20 × 20‐cm square, processed for bulk density and percentage carbon content in the fine fraction that passes through an 8‐mm sieve. Once the organic horizon is sampled, the mineral soil is sampled sequentially downward in the soil profile, down to 55 cm from the top of the mineral soil. However, re‐measurement is only for the 0–15 cm mineral soil layer. This layer is sampled by digging a hole with a trowel and taking its volume with glass beads and a graduated cylinder. The soil is analyzed for bulk density and percentage carbon content in the fine fraction that passes through a 2‐mm sieve. On the occasions where the mineral soil was sampled based on soil horizon instead of standardized depth, we standardized soil properties to 0–15‐cm depth using the NFI method of calculating the proportion of each section in the 0–15 cm depth and taking the weighted average stock (National Forest Inventory 2021b). We note that mineral horizons are treated in the NFI as having carbon concentrations less than 17%. We excluded 102 instances of the 3992 mineral soil samples where the concentrations in the top 15‐cm of mineral soil were greater than 17% from our analysis, but our findings were qualitatively unchanged. More details on the sampling and laboratory analyses can be found in the NFI ground plot documentation (National Forest Inventory 2008).

We note also that during NFI plot measurement, organic and mineral soils are not always sampled at each of the four microplots. Factors that prevented sampling include inaccessibility, dangerous conditions, standing water and no mineral soil. The NFI protocol does not allow for samples to be collected elsewhere if the microplot is not accessible (National Forest Inventory 2008). As such, during the initial measurements, 25% of NFI plots had mineral soil data for all four microplots, and > 70% had data for at least three microplots. At remeasurement, 65% and 86%, respectively, had four or at least three microplots sampled. The soil organic horizon data were collected more often, with 56% and 94% of initial measurements having at least 4 and 3 microplots sampled, respectively. This proportion increased to 83% and 95% for remeasurement. Lastly, we highlight that plans to remeasure soils in NFI ground plots a second time are underway, and we include in our analyses 51 plots that have been sampled three times, that is, the initial sampling and at two remeasurement intervals.

### 2.2 Analysis and Simulations

We analyze soil carbon change across the NFI network by dividing the difference in soil carbon between initial and remeasurement data by the number of years between them, permitting all microplots to be evaluated with a common time interval. We report carbon stock data in Mg C ha^−1^ for the entire organic horizon and for the 0–15‐cm layer of the mineral soil. We report the rate of stock change in Mg C ha^−1^ 10 year^−1^. We conduct analyses separately for the organic and mineral horizons. We note that carbon stocks are derived from three soil properties measured in the NFI network. Specifically, the carbon concentration of the fine fraction soil, its bulk density and the volume of coarse fraction in a sample. We explore how variation and estimated change in carbon stocks can be shaped by spatial and remeasurement variability in these three properties.

We test seven research questions under our two objectives to ask about the reliability of estimated soil carbon change and what design decisions could be made to improve reliability. Specifically, (1) what is the relative magnitude of between‐ and within‐plot variation in soil properties under the NFI protocol; (2) does the minimum detectable difference (MDD) in carbon stocks and rates of change differ between mineral and organic horizons; (3) how does increasing the number of plots or microplots per plot affect the MDD; (4) does grouping plots by expected covariates of soil carbon stock reduce measurement variation and MDD; (5) do sequential estimates of soil carbon change show a directional change; (6) are soil carbon stocks biased at sites with fewer sampled microplots; and (7) can change in percent carbon be used as a proxy for change in total carbon without collecting bulk density data?

#### 2.2.1 Within‐ and Between‐Site Variance

We calculated within‐ and between‐plot standard deviation in soil carbon concentration, fine fraction bulk density, coarse fraction, and carbon stocks. We calculated pooled, as opposed to raw, standard deviations to standardize for differences in the number of microplots sampled at each NFI plot. For these calculations, we used the total number of microplots across all plots (*N*), the total number of NFI plots in our data (*k*; indexed by *i*), the number of microplots at each plot ($n_{i}$; indexed by *j*), and the mean value for each soil property measured at each plot (${\bar{y}}_{i}$). Using this information, we calculated the weighted mean over all plots ($\bar{y}$, Equation 1a), the sum of squares within (SSE, Equation 1b) and between (SSB, Equation 1c), and the effective sample size ($n_{\mathrm{eff}}$, Equation 1d). We used these values to calculate the within (Equation 1e) and between (Equation 1f) standard deviation for each soil property in each horizon at each time point. Because each NFI plot has a maximum of four microplots, the estimated variation between plots also incorporates within‐plot sampling noise. The between‐plot standard deviation is thus corrected for within‐plot sampling noise by subtracting the within‐plot error $\left(\frac{\mathrm{SSE}}{N-k}\right)$, where $n_{\mathrm{eff}}$ accounts for unequal numbers of microplots across plots (Quinn and Keough 2002, section 8.2.1).

(1a) $$\bar{y}=\frac{1}{N}\sum_{i=1}^{k}\left(n_{i}{\bar{y}}_{i}\right)$$

(1b) $$\mathrm{SSE}=\sum_{i=1}^{k}\sum_{j=1}^{n_{i}}{\left(y_{\mathrm{ij}}-{\bar{y}}_{i}\right)}^{2}$$

(1c) $$\mathrm{SSB}=\sum_{i=1}^{k}n_{i}{\left({\bar{y}}_{i}-\bar{y}\right)}^{2}$$

(1d) $$n_{\mathrm{eff}}=\frac{N-\frac{1}{N}\sum_{i=1}^{k}{n_{i}}^{2}}{k-1}$$

(1e) $$\sigma_{\text{within},\text{pooled}}=\sqrt{\frac{\mathrm{SSE}}{N-k}}$$

(1f) $$\sigma_{\text{between},\text{pooled}}=\sqrt{\frac{1}{n_{\mathrm{eff}}}\left(\frac{\mathrm{SSB}}{k-1}-\frac{\mathrm{SSE}}{N-k}\right)}$$

#### 2.2.2 Minimum Detectable Difference

We calculated MDD based on the spatial variation in stock inventories from the first or second measurement and the rate of change in stock, scaled to the average re‐measurement period of 10 years across the NFI. The MDD represents the smallest change that the sampling design and measurement variability allowed us to detect with a specified Type I error rate (*α*, e.g., 95%) and power (e.g., 0.80). The approach builds on change detection in soil monitoring networks (Deluz et al. 2020; Saby et al. 2008) that decompose observed variability into between‐unit and re‐measurement components and propagate these into the expected uncertainty of trend estimates. We use the standard MDD on the stock or rate of change using the formula (de Gruijter et al. 2006):

(2) $$\mathrm{MDD}=\left(z_{1-\frac{\alpha }{2}}+z_{\text{power}}\right)\mathrm{SE}$$

where $z_{1-\frac{\alpha }{2}}$ is the critical value of a two‐sided test at significance level α, $z_{\text{power}}$ is the desired power (e.g., 0.80), and SE is the standard error of the carbon stock or rate of change. The estimation of the standard error for carbon stocks is $\mathrm{SE}=\sqrt{\frac{\sigma_{\text{between},\text{pooled}}^{2}}{k}+\frac{\sigma_{\text{within},\text{pooled}}^{2}}{N}}$. Because MDD is sensitive to the power and significance level, we conducted a sensitivity analysis to demonstrate how MDD changed with different levels (Figure S2).

The hierarchical structure of the NFI network allows us to calculate the standard error of the carbon rate of change, ${\mathrm{SE}}_{\text{rate}}$, through the decomposition of two sources of variability: (1) true between site variation in C change rates and (2) within‐site remeasurement error in estimated differences as follows:

(3) $${\mathrm{SE}}_{\text{rate}}=\sqrt{\frac{\sigma_{\text{between}-\text{plot rate}}^{2}+\bar{\sigma_{\text{within}-\text{plot rate}}^{2}}}{m}}$$

where $\sigma_{\text{between}-\text{plot rate}}^{2}$ is the between‐site variance in change, $\bar{\sigma_{\text{within}-\text{plot rate}}^{2}}$ is the average within‐site re‐sampling variance across all sites, and *m* is the number of paired microplots.

The between‐site variance contains both true differences between sites and measurement error, which we assume to be independent because we only have a single dataset from which to estimate them. So, the between‐site rate is calculated by subtracting the observed between‐site variance by the average within‐site variances. This value cannot be less than zero (Saby et al. 2008):

(4) $$\sigma_{\text{between}-\text{plot rate}}^{2}=\max\left(0,\sigma_{\text{observed between}-\text{plot rate}}^{2}-\bar{\sigma_{\text{within}-\text{plot rate}}^{2}}\right)$$

The observed between site variance ($\sigma_{\text{observed between}-\text{plot rate}}^{2}$) is calculated using the following formula:

(5) $$\sigma_{\text{observed between}-\text{plot rate}}^{2}=\mathrm{Var}\left(\frac{Y_{1,i}-Y_{0,i}}{T_{1}-T_{0}}\right)$$

where $Y_{1,i}$ and $Y_{0,i}$ are the site‐level mean C stocks at times $T_{1}$ and $T_{0}$, respectively.

The variance of within‐site change is calculated as (de Gruijter et al. 2006, equation 15.20),

(6) $$\sigma_{\text{within}-\text{plot change},\;i}^{2}=\frac{v_{1,i}}{n_{1,i}}+\frac{v_{0,i}}{n_{0,i}}-2\rho \sqrt{\frac{v_{1,i}}{n_{1,i}}\times \frac{v_{0,i}}{n_{0,i}}}$$

where $v_{0,i}$ and $v_{1,i}$ denote the within‐site variance at time 0 and 1, with the number of microplots $n_{o,i}$ and $n_{1,i}$ within the site and ρ is the correlation between paired microplots. Then, this is used given that k is the total number of i sites, to calculate the average within‐site variance in change ($\bar{\sigma_{\text{within}-\text{plot rate}}^{2}}$) as the mean of the within‐site variance of change for each site standardized by re‐measurement interval:

(7) $$\bar{\sigma_{\text{within}-\text{plot rate}}^{2}}=\frac{1}{k}\sum_{i=1}^{k}\frac{\sigma_{\text{within}-\text{plot change},i}^{2}}{{\left(T_{1}-T_{0}\right)}^{2}}$$

Throughout, we use the rate of change over 10 years to represent the MDD of the network but note that because the interval between remeasuring NFI sites varied, the resulting MDD should be interpreted cautiously. A longer period between re‐measurement decreases the magnitude of rate estimates due to an increasing denominator (∆T), which can systematically alter the relative weight of a site that was remeasured after 5 years compared to 15 years. Our MDD for stocks, also calculated using Equation (2), are unaffected by this potential bias.

#### 2.2.3 Sample Number and MDD

We conducted a simulation study to change the number of microplots sampled per plot and the number of plots sampled. We used the empirical within and between average standard deviation of 17.3 and 19.0 Mg ha^−1^ in soil carbon stocks to simulate a T0 dataset. For each site, a change of 2.3 Mg ha^−1^ over 10 years with a between‐site standard deviation of 18.4 Mg ha^−1^ was used. This change and variance estimate is based on the dataset mean rate of change estimate, 0.23 Mg ha^−1^ year^−1^ for a 10‐year period. We varied the number of microplots from 2 to 8 and the number of NFI plots sampled from 1 to 1000.

#### 2.2.4 Grouping Plots

We recalculated the within‐ and between‐plot standard deviations for each ecozone in the NFI dataset. We chose ecozone as our grouping variable because ecozones are meant to capture large clusters of similar environments with comparable climate conditions and plant communities (Wiken 1986). We consider whether clustering the NFI plots by ecozone reduces the between‐plot standard deviation—when controlling for sample size—therefore reduces the MDD (de Gruijter et al. 2006).

#### 2.2.5 Directional Change in Time

Our fifth question required at least two re‐measurements, which restricted us to the subset of 51 NFI plots where soil properties were measured three times. We calculated the rate of change in soil properties over the first and second intervals and plotted them against each other. Analysis of repeated measurements of change can help to identify the extent to which estimates of change reflect real change or spurious estimates caused by sampling a spatially variable property. The emergent phenomenon, known as regression to the mean, arises when measures at the tails of a distribution at time 0 lead to large positive and negative estimates of change simply because the time 1 measurement is more likely to be closer to the true mean (Lark et al. 2006). A strong positive correlation between rates of change in the first and second intervals would suggest that directional change is real and not due to sampling variation.

We also used the approach of Lark et al. (2006) to test for an effect of initial stock size. A negative correlation between the rate of change and the first measurement indicates regression to the mean. Lark et al. (2006) argue that if there is also a negative correlation between the rate of change and the average of the first and second measurement, this is evidence of a rate of change dependent on initial stock size instead of being an artifact of regression to the mean.

#### 2.2.6 Microplot Sampling Bias

We used a generalized linear mixed effects model to estimate the effect of the number of microplots sampled on the average plot level soil carbon. We included measurement event as a fixed effect and the NFI plot identity as a random intercept to account for the repeated temporal measurements at each NFI plot and the repeated spatial measurements of multiple microplots at each NFI plot. We used a gamma distribution with a logarithmic link and added extra dispersion parameters for the number of microplots and the measurement round to improve the model fit. Using the site‐level average may bias the analysis because plots with more microplots might have less variation. To test the robustness of our results, we repeated our analysis 100 times by randomly drawing one microplot to represent the site average to check if our model coefficients remained stable.

#### 2.2.7 Percent Carbon as a Proxy

We plotted the relationship between the change in total carbon and the change in percent carbon across all the NFI plots for both the organic and mineral horizons. We calculated the $R^{2}$ value for these relationships as a measure of agreement between these two estimates.

### 2.3 Software

We conducted our analysis using R (version 4.5.0) (R Core Team 2025). We used the packages tidyverse (version 2.0.0), lubridate (version 1.9.4), glmmTMB (version 1.1.11), and lme4 (version 1.1‐37) (Bates et al. 2011; Grolemund and Wickham 2011; McGillycuddy et al. 2025; Wickham et al. 2019). We used suggestions from Microsoft Copilot (version GPT‐5) to improve our code and manuscript text.

## 3 Results

### 3.1 Within‐ and Between‐Site Variance

**FIGURE 2:**
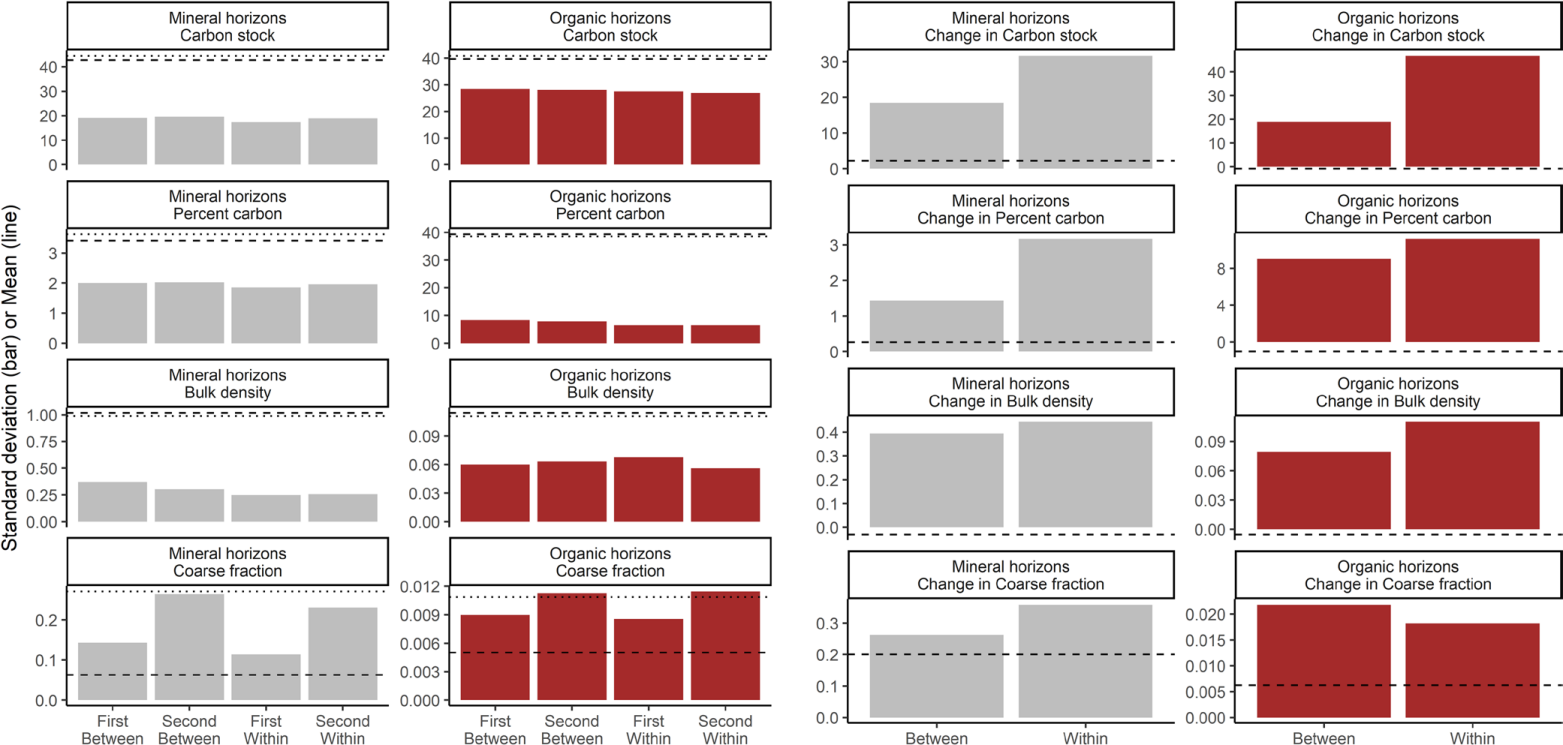
Measured spatial variation within and between sites and measured temporal variation in change in soil carbon stocks, % carbon, fine fraction bulk density and coarse fraction bulk density for organic and mineral horizons. Fine fraction bulk density for the mineral soil is calculated as g cm^−3^ subtracting an estimate of coarse fraction volume. Coarse fraction bulk density and fine fraction bulk density for the organic horizon is g cm^−3^ of the sampled volume, because for the organic horizon, coarse fraction contributes to the carbon stock. Carbon stocks are reported in Mg C ha^−1^ and change is Mg C ha^−1^ 10 year^−1^. Mean values for each variable are shown as horizontal lines, with the first (*t*_0_) measurement as a dashed line and the second (*t*_1_) as a dotted line for the inventory estimates (left two columns) and as a dashed line for the change estimates (right two columns).

Between‐ and within‐site variation in soil carbon properties were large (Figure 2). Between‐site standard deviation in soil carbon stock was 44% and 70% of the mean value for the mineral and organic horizons, respectively. Within‐site variation was only slightly lower at 41% and 68%, making it relatively comparable in magnitude to between‐site variation (Figure 2). For the soil properties from which stocks were calculated, the organic horizon had lower standard deviation in carbon concentration than the mineral horizon, whereas the latter had lower and more consistent variation in bulk density. Coarse fraction variation was commonly high (Figure 2).

Between‐ and within‐site standard deviation in the rate of change in soil carbon properties was much larger than the mean estimates (Figure 2). Rates of change were 2.3 and −0.867 Mg ha^−1^ 10 year^−1^, whereas between‐site standard deviation for change in soil carbon stocks was 18.5 and 18.9 Mg ha^−1^ over a 10‐year period for mineral and organic horizons, respectively. Within‐site standard deviation was larger at 31.5 and 46.7 Mg ha^−1^ 10 year^−1^, respectively. Changes in percent carbon were relatively more variable in the organic horizon, while changes in bulk density and coarse fraction content were more variable in the mineral horizon (Figure 2).

### 3.2 Minimum Detectable Difference

**FIGURE 3:**
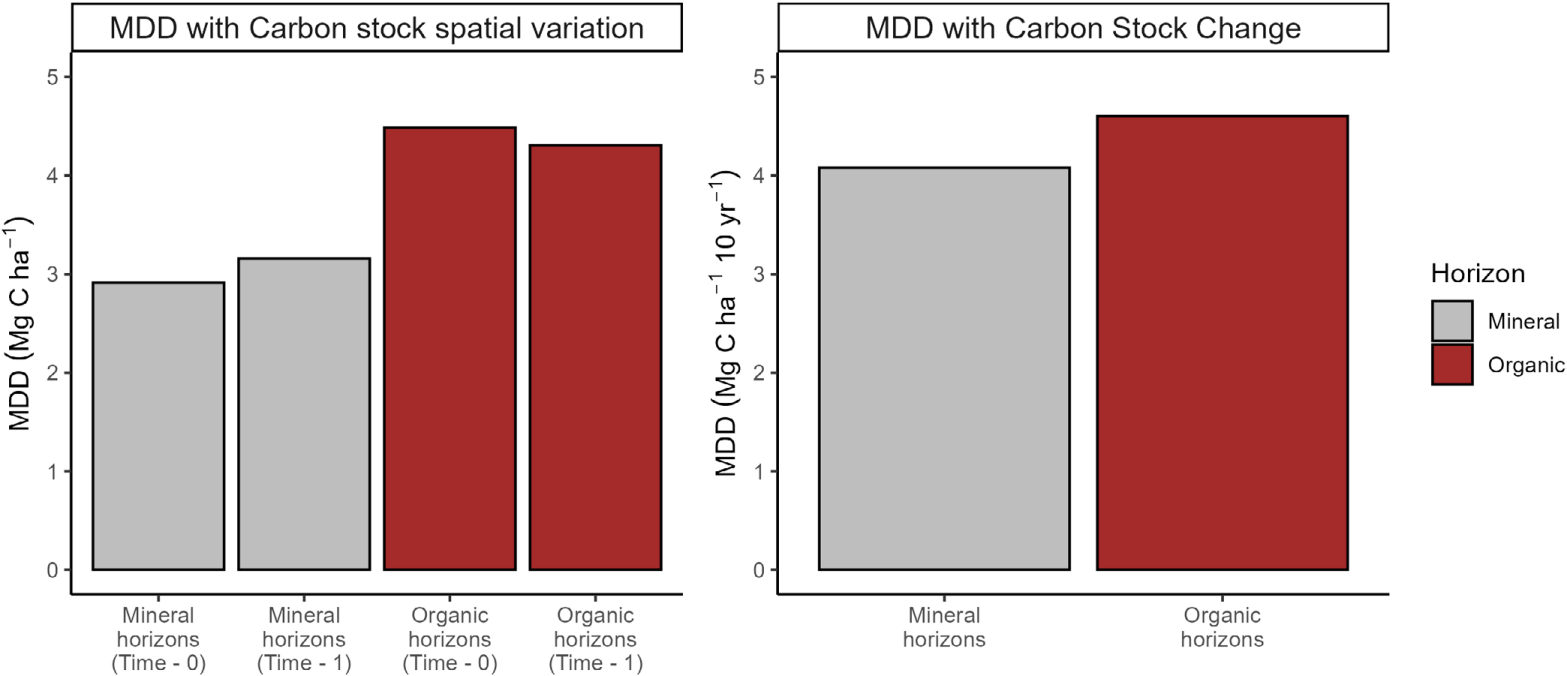
The minimum detectable difference (MDD) in soil carbon stocks and rate of change using the National Forest Inventory remeasurement data set. The left facet calculates MDD using the spatial variation from the first measurement and the second one separately; the right facet calculates the MDD of change over a 10‐year period using the variation in rates of change of soil carbon stocks between the first (i.e., initial) and second (remeasurement) times.

The MDD for the soil organic horizon stock of ~4.4 Mg ha^−1^ was larger than the MDD for the mineral soil horizon of ~3 Mg ha^−1^ (Figure 3). MDD ranged from 2 to 7 Mg ha^−1^ and 3 to 7 Mg ha^−1^ 10 year^−1^ for organic and mineral horizons when significance level and power were varied from 0.001 to 0.15 and 0.6 to 0.9, respectively (Figure S1). When the MDD was instead calculated using change in stocks from initial to remeasurement, as opposed to initial stock values, the MDD values for the organic and mineral horizons were 4.61 and 4.08 Mg ha^−1^ 10 year^−1^ (Figure 3).

### 3.3 Sample Number and MDD

**FIGURE 4:**
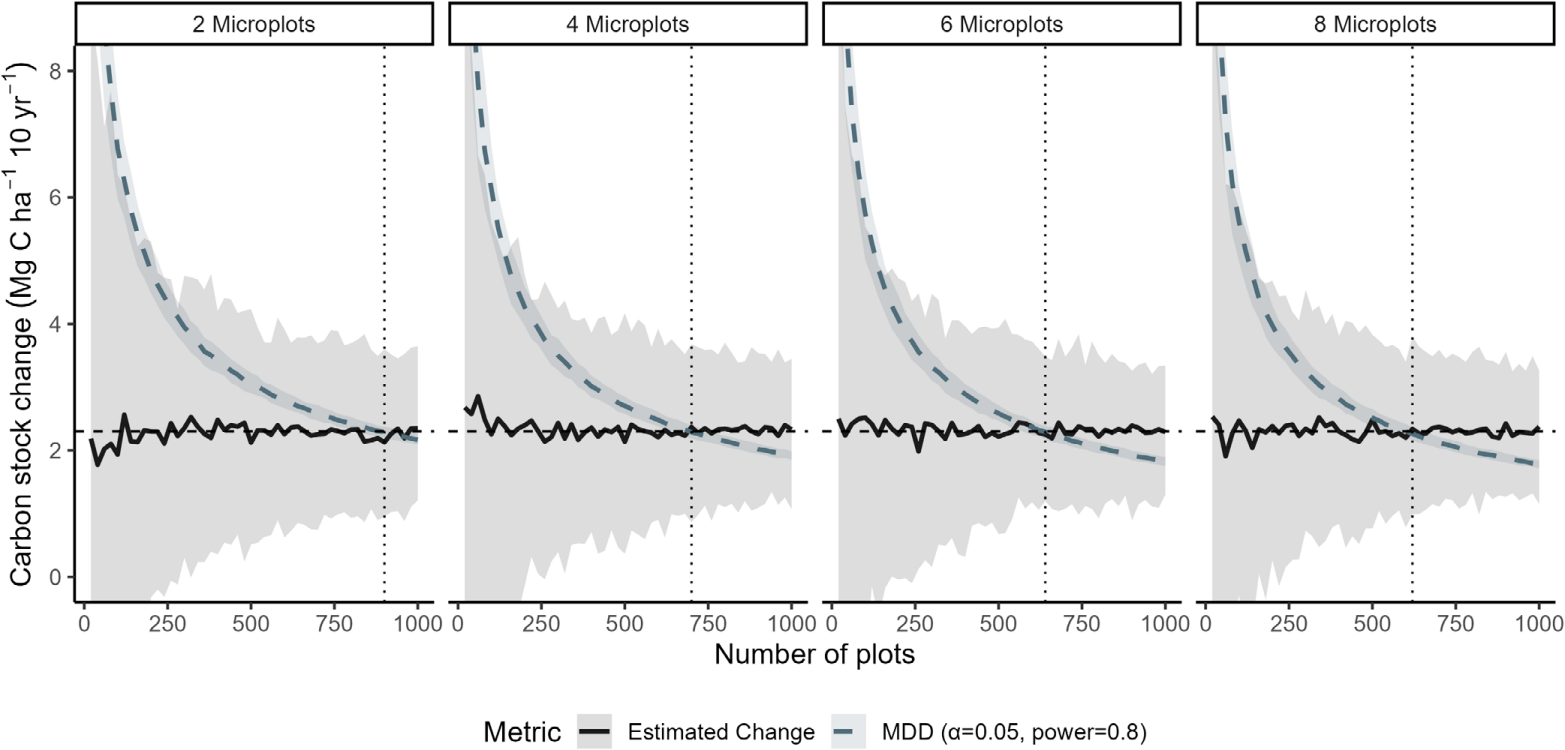
Using the within‐ and between‐plot variance in C stocks from the mineral horizons dataset (17.3 and 19.0 Mg C ha^−1^) and a 2.3 Mg C ha^−1^ 10 year^−1^ change, we simulate how the Minimum Detectable Difference (MDD) responds to increasing the number of sites (*x* axis) or plots per site (facets). The simulations show the range in estimates from re‐sampled datasets on the estimated stock change from the NFI remeasurement (black line and boot‐strapped 95% confidence interval) and the MDD (blue‐grey dashed line). Within a simulated set of microplots per site, the vertical line denotes the number of sites after which the study has 80% power.

Our simulations demonstrated a clear benefit of increasing the number of microplots per plot or the number of plots sampled (Figure 4). For example, for the MDD to be below the empirical rate of change of ~2.3 Mg ha^−1^ 10 year^−1^ estimated from the initial and remeasurement of the mineral horizon stocks, required ~900 plots with two microplots or ~620 plots with eight microplots. Note that the impact on lowering the MDD with increasing plot or microplot numbers decreases with increasing sampling density where, for example, for the MDD to approximate 2.3 Mg ha^−1^ 10 year^−1^ requires 700, 640 or 620 sites with 4, 6 or 8 microplots (Figure 4), respectively, and site number only drops to 580 with 10 microplots (not shown for visual clarity of Figure 4).

### 3.4 Grouping Plots

**FIGURE 5:**
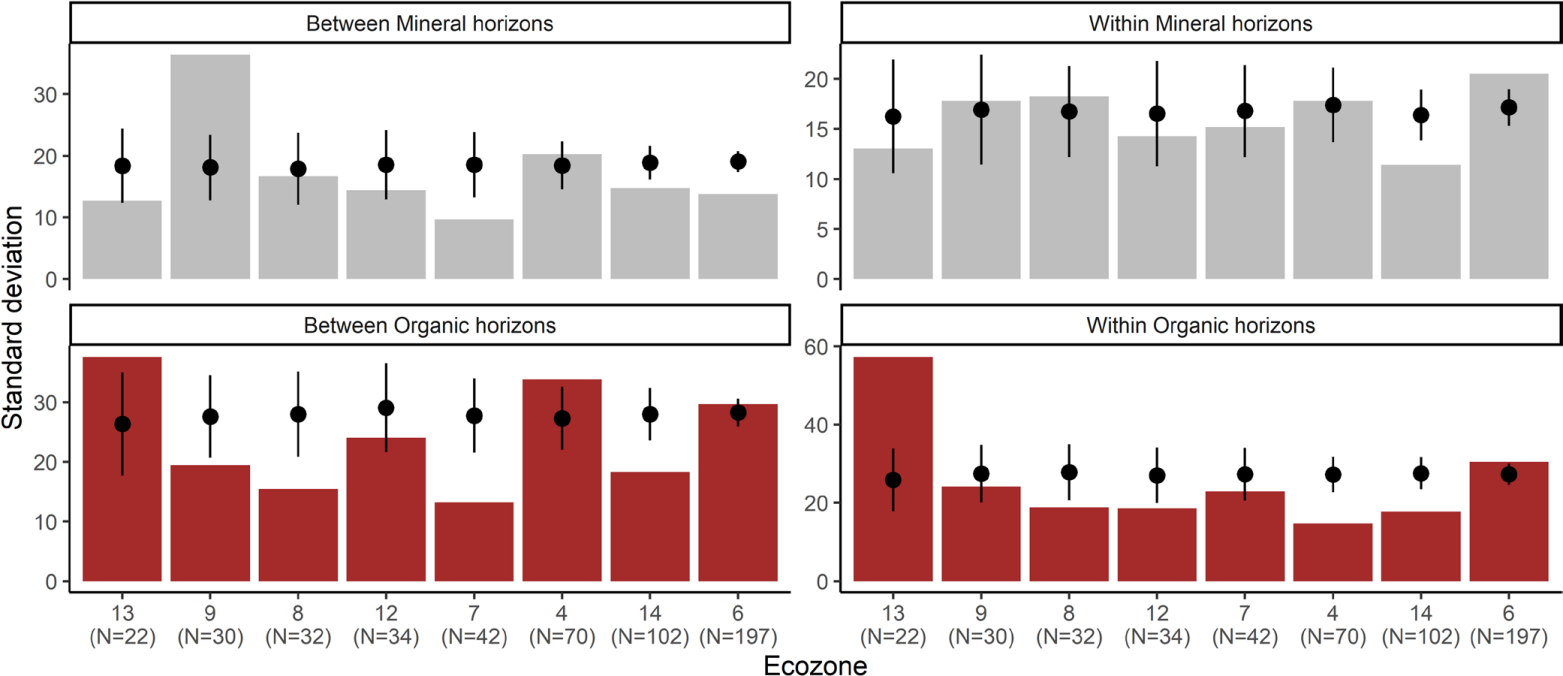
Between‐ and within‐plot standard deviation in soil carbon stocks across ecozones, which are ordered from left to right based on the number of plots sampled in each (shown below the ecozone number on the x‐axes). Bars show the value for each ecozone, while points show the standard deviation for the same number of plots selected at random from across the plot network. Error bars around the points summarize 100 draws of these randomly selected plots. The ecozones are: Pacific Maritime (13), Boreal Plains (9), Mixedwood Plains (8), Boreal Cordillera (12), Atlantic Maritime (7), Taiga Plains (4), Montane Cordillera (14), and Boreal Shield (6).

Grouping plots by ecozone was sometimes effective at reducing between‐site variation in soil carbon stocks (Figure 5). The biggest reductions in between‐plot standard deviation for the mineral stocks, compared with an equal number of randomly selected NFI plots, were seen for the Atlantic Maritime (7), Montane Cordillera (14), and Boreal Shield (6) ecozones. Yet stocks were more variable across plots in the Boreal Plains (9). For the organic horizon, improvements (i.e., reductions in variation) were observed for the Mixedwood Plains (8), Atlantic Maritime (7), and Montane Cordillera (14), but variation was greater—again compared to random draw of NFI plots, for the Pacific Maritime (13) and Taiga Plains (4) ecozones. We observed similar outcomes of grouping plots for within‐plot standard deviation (Figure 5), with some ecozones being less variable and others more variable when compared to the national network, suggesting grouping is not a universal solution to addressing between‐ and within‐plot variation.

### 3.5 Directional Change in Time

**FIGURE 6:**
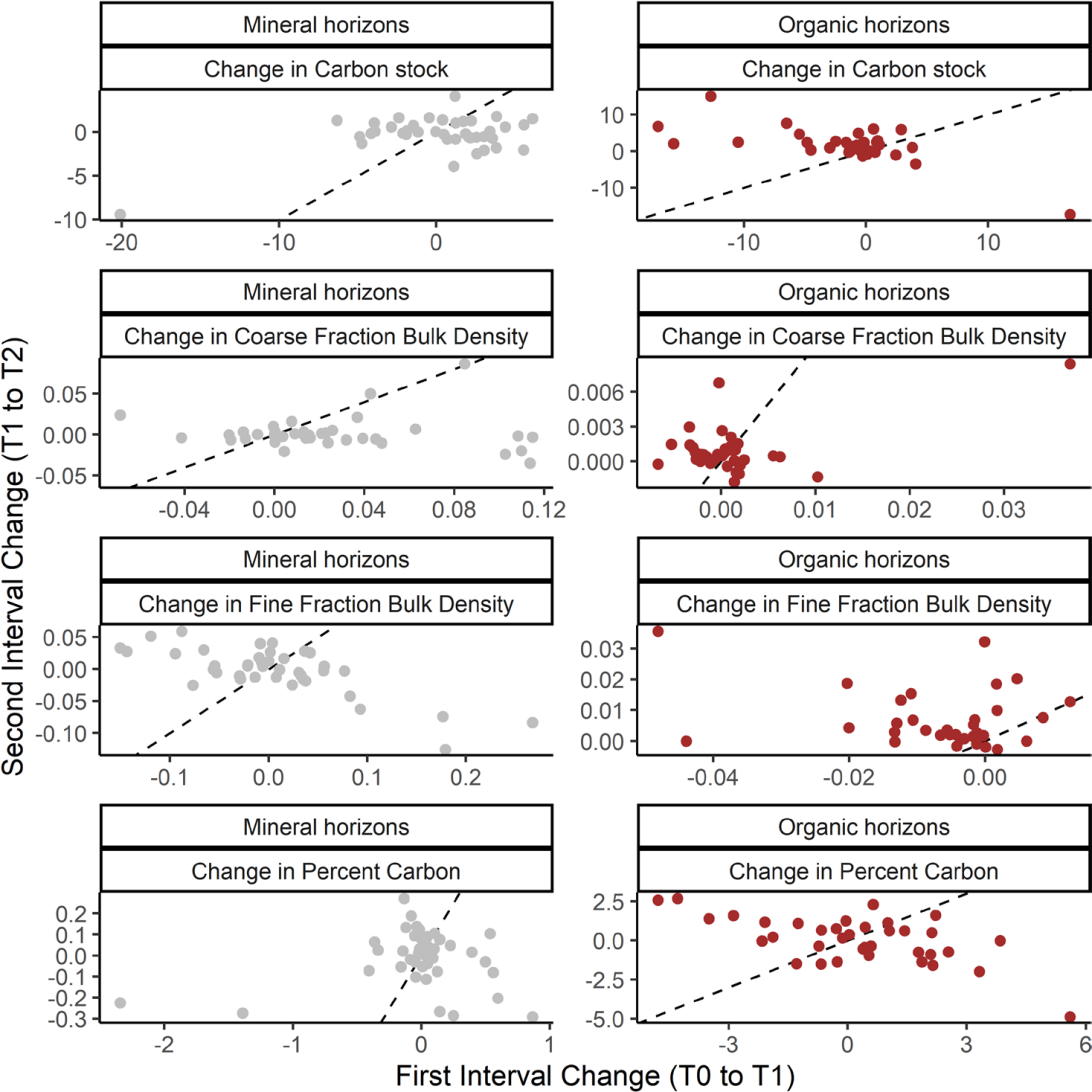
The relationship between change in soil carbon properties over the first (T0 to T1) and second (T1 to T2) intervals across all NFI plots where at least three temporal measurements have been taken (*N* = 51). Generally, T0 occurs in the 2000s, T1 in the 2010s, and T2 in the 2020s. Each point represents the change at the plot level averaging all available paired microplots. The rows indicate different soil properties, while the columns are mineral (left) and organic (right) horizons. A positive correlation on or parallel to the dashed line indicates consistency of the estimated directional change over the two sampling intervals. The lack of a relationship suggests that the estimate of change in the first interval likely reflected the statistical phenomenon of regression to the mean.

Organic and mineral soil carbon stocks displayed an emergent pattern, referred to as regression to the mean, whereby the change in total soil carbon from initial measurement to the first remeasurement was negatively correlated with the initial soil carbon stock (Figure S3). We tested whether this pattern was genuine or a statistical phenomenon using two different approaches. First, the NFI has some plots that have been remeasured a second time. True directional change would be likely to continue from the first to second remeasurement, but we found the interval 1 and 2 rates of change were not correlated (Figure 6). We note also that the three soil properties used to determine stocks also showed regression to the mean patterns and had non‐correlated interval 1 to 2 change values (Figure 6). Yet because NFI plots remeasured to calculate a second interval only numbered 51, we also tested for regression to the mean using the full dataset. Regressing change against the mean stock values for the initial and first remeasurement values nullified the negative relationship between estimated change and the initial stock measurement (Figure S3), further suggesting that the change we estimated was likely a statistical artifact of sampling spatially variable data.

### 3.6 Microplot Sampling Bias

The mineral soil carbon stock at an NFI plot was higher on average in locations where only one or two microplots were sampled relative to plots where three or four microplots were sampled (Figure S4). On average, adding a microplot decreased the estimated mineral soil carbon by ~1.5 Mg ha^−1^. This trend was not always significant when we randomly selected one microplot to represent the NFI plot mean, instead of taking the mean of a different number of microplots. However, the direction and magnitude of the effect was consistent across these random iterations, suggesting systematic bias in stock estimates where only one or two microplots were sampled. The systematic bias in stocks appeared to emerge from a systematic bias in soil carbon concentration, which decreased by a value of ~0.3% with each added microplot. The effect of this decrease on stocks was countered, in part, by a relatively smaller increase in bulk density of ~0.04 g cm^−3^ per added microplot. In contrast to the mineral horizon, there was no apparent systematic bias of microplot number sampled on the organic horizon carbon stock (Figure S4).

### 3.7 Percent Carbon as a Proxy

**FIGURE 7:**
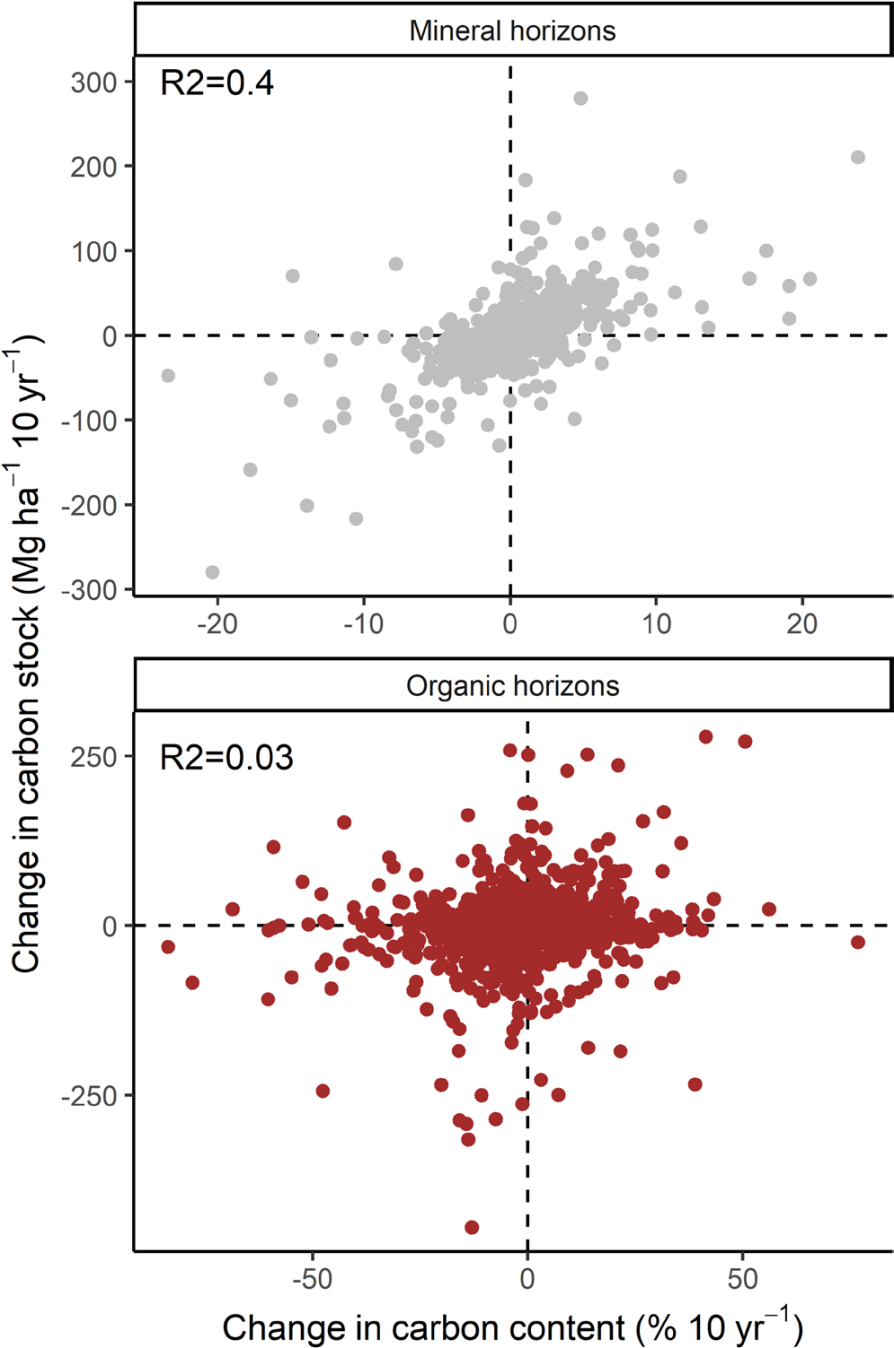
The relationship between change in carbon concentrations and change in carbon stocks for the mineral and organic horizons. The reported *R* ^2^ is from a linear mixed effects model with plot as a random effect to account for the measurement of change in multiple microplots within the same plot. The relationships are relatively weak, with marked variation from the mean line (mineral horizon) or absent (organic horizon).

Given suggestions that changes in soil carbon concentrations might serve as a suitable proxy for changes in soil carbon stocks (Walter et al. 2016; Zouhar et al. 2024), we evaluated the relationship for the mineral and organic horizons. There was a correlation for the mineral but not organic horizon (Figure 7). Yet percentage carbon change in the mineral horizon only explained 40% of the variation in stock change, suggesting that there is meaningful variation attributable to bulk density (and/or coarse fraction) changes between remeasurements. The same trend was observed for the relationship between percent carbon concentration and carbon stocks (Figure S5).

## 4 Discussion

National and multi‐national soil monitoring networks have the potential to inform coupled carbon cycle‐climate models and large‐scale policy efforts to mitigate losses of ecosystem carbon stores. However, carbon stocks in forest soils are notoriously variable at local to regional scales, creating uncertainty about the ability of monitoring networks to detect and quantify stock changes (Lang et al. 2025; Schrumpf et al. 2011). We used the Canadian NFI soil carbon dataset, which presents a relatively unprecedented amount of re‐measurement data of forest plots arrayed across a national scale. We use the NFI dataset to learn about how design decisions, such as the number of plots, grouping, and within‐plot sampling density, might affect the effectiveness of such networks to reliably quantify change (summarized in Table S1).

We found high within‐ and between‐plot variation in soil carbon stocks and those properties used to calculate it. Comparable studies in grassland or cropping systems have found coefficients of variation (CV) of ~9% (Poeplau et al. 2022), 4% (Saby et al. 2008), 10%–24% (Conant and Paustian 2002), and up to 30% (Goidts et al. 2009) in mineral soil carbon stocks. A review found values for grasslands ranging from 0.5% to 89% with a mean of 22% (Maillard et al. 2017). We found values for the top 15‐cm mineral soil in Canadian forests of ~40%. Variation in the organic horizon was even larger (CV ~70%). Revealing just how pronounced local variation is, our estimates of within‐plot variability are similar to those for between‐plot variability. Such high variability may be a common feature of forest soil carbon stocks because of high coarse fragment content, large organic and mineral horizon stocks, and variable organic matter inputs from a mixture of plant functional types (Lang et al. 2025; Schrumpf et al. 2011). For example, in European forests, the CV for mineral soils was comparable to the NFI and, although values for the same forest soils were closer to 50% for the organic horizons (Schrumpf et al. 2011). Overall, the CV for soil carbon stocks in cold temperate and boreal forests appears much larger than for arable and grassland cover types. The higher variation we observed for the organic horizon in Canadian forests may reflect the much larger range of forest types included in the NFI dataset relative to previous work and the high within‐plot variability that exists in the Pacific maritime and Boreal shield ecozones. Regardless of the reasons for the high variation that we observed for the organic horizon, the similarity of our CV for the surface mineral soil to other forest soil assessments (Schrumpf et al. 2011) meant that the MDD values of ~3.0 Mg ha^−1^ from the spatial inventory data match those for surface mineral soils in European forests (Schrumpf et al. 2011).

The challenge with using spatial MDD estimates is that, in monitoring networks, what we want to know is our ability to detect change in time. Using the remeasurement data, the temporal MDD estimate for the mineral soils was about 1–1.5 Mg ha^−1^ larger than the spatial MDD values in our and other studies (Schrumpf et al. 2011). Specifically, we found that with the current sampling effort the NFI program in Canada can likely detect changes—at least under the assumptions used to calculate MDD—in forest carbon stocks of ~4.1 Mg ha^−1^ 10 year^−1^ for the top 15‐cm of the mineral soil. The higher temporal versus spatial MDD estimates likely reflect the compounding of uncertainties, such as in remeasurement variation (Potash et al. 2025). Notably, the NFI program covers Canada's productive—or working—forests and so can be used for national‐level greenhouse gas inventories. The temporal MDD estimate for mineral soils suggests the current data could be used to detect 1.5 Pg, or more, of net carbon lost (or gained) from surface mineral soils across the country over 10 years. By contrast, Canada's total estimated forest C emissions in 2023 were estimated to be 0.0065 Pg (Environment and Climate Change Canada 2025). As such, it is unlikely that the current NFI design would be able to detect changes in national forest soil carbon stocks unless they were about two orders of magnitude above current annual emissions (Saby et al. 2008).

The temporal MDD change estimate for the mineral soil horizon was higher than the actual estimated rate of change in mineral soil carbon stocks across the NFI. Specifically, our analysis of the NFI data suggested mineral carbon significantly increased by 0.23 Mg ha^−1^ year^−1^ (*p* = 0.047). Given recommendations to remeasure forest soil carbon every 10 years (Schrumpf et al. 2011), this rate corresponds to an increase of 2.3 Mg ha^−1^ 10 year^−1^. The magnitude of estimated change is comparable to mean estimates of soil carbon gain, or avoided emissions, under adoption of regenerative agricultural practices and is considered meaningful for climate mitigation (Blanco‐Canqui 2022; Bolinder et al. 2020; Poeplau and Don 2015; Sanderman and Baldock 2010). However, given that the temporal MDD for the network was estimated at about twice this value (4.1 Mg ha^−1^ 10 year^−1^), it is possible that the rate of gain is overestimated. Specifically, this rate of change may reflect the Winner's Curse, which is a phenomenon whereby statistically significant effects from underpowered studies overestimate the true effect to reach significance (Button et al. 2013; Van Zwet et al. 2023). However, significance—and relatedly MDD—rely on assumptions that have been repeatedly criticized as the basis for determining whether an estimated effect approximates reality (Goodman 1999; Greenland et al. 2016; Wasserstein et al. 2019). A strength of the Canadian NFI then is the intention to continue remeasurement, because change estimates emerging from statistical phenomena caused by sampling variation are unlikely to be directionally consistent across repeated resampling intervals.

Fifty‐one plots of the NFI had microplot resampling data available for a second remeasurement of soil carbon stocks. Our analysis of these data suggested that the change of 2.3 Mg ha^−1^ 10 year^−1^ estimated from the first remeasurement was not sustained for a second 10‐year period. Indeed, none of the change estimates for the three soil properties used to calculate stocks were correlated between the first and second remeasurement intervals. However, given that only ~10% of plots had this second remeasurement, future analyses using the full third measurement data, when they become available, will be necessary to build confidence that there is no directional change in forest soil carbon stocks. Nevertheless, the first remeasurement data displayed patterns that are consistent with strong emergence of a statistical phenomenon called regression to the mean, which can lead to erroneous conclusions about stock change in soil carbon datasets (Bradford et al. 2023; Slessarev et al. 2023). This pattern does not necessarily confirm that change estimates are an artifact (Bellamy et al. 2005) but, when we regressed change against the mean stock values for the initial and first remeasurement values, the patterning was also nullified. Such an approach has been proposed to diagnose whether an effect is real or statistical artifact (Lark et al. 2006). Based on our analyses, we suggest that soil carbon change estimated by the NFI should be interpreted with caution at this time but equally that the network is designed in a way—with continuing remeasurement—that it will likely be able to separate spurious change estimates from those that are repeatable and hence more reliable. As such, our findings emphasize the value of repeated remeasurement for soil monitoring networks in general, to separate spurious from directional change in soil carbon. Although there is some disagreement about the necessary temporal frequency of such remeasurement (Harbo et al. 2025; Nerger et al. 2020), for national‐scale networks it may be that decadal sampling—as in the NFI—provides an appropriate balance between economic feasibility and confident detection of change (Goidts et al. 2009; Harbo et al. 2025; Saby et al. 2008).

Our simulations demonstrate the current strengths and potential improvements to the Canadian NFI network's design for reliably detecting and quantifying changes in organic and mineral soil carbon stocks. First, plans for multiple remeasurements will generate confidence that estimates of soil carbon stock change are real and not a statistical artifact. Second, the temporal MDD values suggest that if all the 801 ground plots in the network are remeasured then, with four microplots, changes of ~2 Mg C ha^−1^ 10 year^−1^ should be detectable. Increasing the number of microplots would reduce the number of plots needed to detect this change, albeit the benefit of increased sampling numbers tails off quite rapidly, suggesting that the greatest benefit can likely be achieved through only modest increases in plot or microplot numbers. Other monitoring assessments reveal that more per‐site samples correspond with the need for a smaller number of sites to robustly detect change (Bradford et al. 2023; Goidts et al. 2009; Poeplau et al. 2022; Schrumpf et al. 2011). However, the value of maintaining the same design for long‐term networks such as the NFI may instead suggest that, for the Canadian NFI, adding plots—as opposed to microplots—would be the preferred strategy. For newly established forest monitoring programs, our simulations indicate that somewhere between 4 and 6 microplots is a reasonable balance between effort and sampling to address within‐plot variation.

Adding more remeasurement plots to the Canadian NFI network would likely increase its power to detect ecozone‐level changes in soil carbon. Such sub‐group analyses might be expected to permit detection of smaller soil carbon change by decreasing the variation between plots within each ecozone. To test this assumption, we calculated the between‐ and within‐plot standard deviation—which directly relates to MDD—for each ecozone in Canada. We found modest and inconsistent reductions in between‐plot standard deviation for some ecozones. Specifically, the plains' ecozones have high variation in soil types and soil carbon stocks (Wiken 1986), with the consequence that adding plots to ecozones such as the Boreal, Taiga, and Mixedwood Plains in Canada may be particularly effective for addressing ecozone‐level uncertainty and hence improving the capabilities of the NFI to detect meaningful change in stocks. More generally, our results suggest that ecozones with more variable soil carbon stocks will require more sampling in new or existing monitoring programs to make subgroup analyses robust.

Even if adding plots is the preferred strategy, our analysis does suggest that measuring fewer than the current four microplots may be associated with systematic bias in plot‐level stock estimates. That is, we found that plots with fewer microplots sampled tended to have higher mineral soil carbon stocks. The reason(s) for this bias cannot be definitively ascertained from the NFI data but sampling < 4 microplots was an outcome of inaccessibility or dangerous conditions associated with those unsampled microplots. What is certain from our simulations is that measuring < 4 microplots is associated with large increases in MDD. It may then be advantageous to add to these existing plots new microplots in safe, accessible locations to standardize sampling effort to 4 microplots per plot.

Even without the addition of plots to the network, or changes to guarantee that 4 microplots are sampled per plot, our analysis suggested the potential for the Canadian NFI to detect widespread change in soil carbon as long as it is large enough. For example, disturbances such as high‐intensity wildfire and invasion of non‐native earthworms can cause almost complete loss of the organic horizon that would be detectable if widespread across the network (Buchkowski et al. 2024; Cameron et al. 2015; Yi et al. 2010). In contrast, the effects of forest harvesting on mineral soil carbon stocks might barely be detectable given that current estimates suggest a loss of less than 10% (Mayer et al. 2020; Sanderman et al. 2017), where our MDD is ~8%. The chances of detecting a change in organic horizon soil carbon stock after harvesting would be higher since the decline is expected to be ~30% (Mayer et al. 2020). Similarly, our analysis suggests that the current NFI could not detect soil carbon gains or loss associated with widespread afforestation (Mayer et al. 2020). We find that the Canadian NFI network is well designed for detecting impacts of some disturbances and management on soil carbon losses at the scale of national, productive forest in Canada, but not powerful enough to detect the early accumulation of losses or gains.

Soil carbon monitoring can also benefit from integration with remotely‐sensed data products and process‐based models to help explain spatial and temporal variability (e.g., Hararuk et al. 2017; Sothe et al. 2022). We chose to focus on empirical, ground plot data because our questions were about accurate field measurement, which is the first step toward building a robust dataset that is useful for evaluating process‐based and digital soil‐mapping models (Bradford et al. 2025). Specifically, measurement and remeasurement data sets, such as the Canadian NFI, are critical for developing and proving process‐based and digital mapping models as approaches suited to large‐scale, soil carbon accounting.

Overall, Canada's NFI soil sampling program provides a unique dataset—given its measurement of multiple microplots per plot and extensive remeasurement data—for assessing design choices that improve the ability to detect and reliably quantify soil carbon change in forests at regional to national scales. To measure changes in stocks as accurately as possible would require remeasurement of deeper soil depths and the data necessary to compare carbon stocks based on equivalent soil mass (Goidts et al. 2009). Nevertheless, the measurement of multiple soil properties that underpin stock calculations affords insights into directional changes in soil conditions. Our analysis suggests that increased investment in forest soil carbon monitoring in Canada, through remeasurement of all ground plots and potentially the addition of some plots in more variable ecozones, could produce an even more robust dataset that would enable the detection of smaller national and potentially regional changes that may occur in response to climate change, the spread of non‐native earthworms, and increased wildfire. At a minimum, the continued collection of this dataset will provide important insights into how the large, heterogeneous, and ecologically important pool of carbon stored in surface forest soils is changing under simultaneous factors such as climate change and forest management.

## Author Contributions

**Alexander Polussa:** conceptualization, writing – review and editing, visualization, methodology, formal analysis, validation. **Robert W. Buchkowski:** conceptualization, writing – original draft, methodology, validation, visualization, formal analysis. **Mark A. Bradford:** conceptualization, writing – review and editing, validation, methodology.

## Funding

R.W.B. was generously supported by the Natural Science and Engineering Research Council of Canada and the Canada Research Chairs program (Grant Numbers: RGPIN‐2024‐05238 & CRC‐2023‐00334). A.P. and M.A.B. conducted the work as part of the Yale Applied Science Synthesis Program, with support from the Yale Center for Natural Carbon Capture, the Environmental Defense Fund.

## Conflicts of Interest

The authors declare no conflicts of interest.

## Supporting information


**Table S1:** The seven central questions of our analysis and answers to them based on analyzing Canada's National Forest Inventory Data.
**Figure S1:**. The carbon stock across mineral and organic horizons by sampling month to demonstrate that the time of the snow‐free season in which a sample was collected did not lead to a systematic bias in the carbon measurement in the Canadian National Forest Inventory Dataset.
**Figure S2:** The minimum detectable difference (MDD) in soil carbon stocks and rate of change using the National Forest Inventory remeasurement data set across different choices for significance level (α; left column) and statistical power (right column). The top row reports MDD using the spatial variation from the first measurement and the second one separately; the bottom row reports the MDD of change over a 10‐year period using the variation in rates of change of soil carbon stocks between the first (i.e., initial) and second (remeasurement) times. Figure 3 in the main text corresponds to α = 0.05 and power = 0.8.
**Figure S3:**. Regression to the mean patterns for changes in carbon stock across all the National Forest Inventory plots (A). Points represent a plot mean (across microplots) for the initial carbon stock and change in total carbon stock between the initial and first remeasurement (*N* = 532 plots). (B) The relationship between mean carbon stock, calculated from the initial and first remeasurement data, adjusted linearly to 10‐years after the first sample. The disappearance of the negative relationship in A is suggestive of the relationship in A being a statistical artifact of sampling a spatially variable outcome, which gives rise to a pattern referred to as regression to the mean.
**Figure S4:**. The difference in total soil carbon stock between plots with different numbers of microplots. The cyan diamonds show the mean values. Statistical significance was evaluated with a generalized linear mixed effects model with plot as a random effect to account for multiple measurements per plot. For the mineral horizon, mean total carbon decreased as the number of microplots increased with plots with one microplot having 5.6 Mg ha^−1^ more carbon than the average plot.
**Figure S5:**. The relationship between carbon concentrations and stocks for the initial measurement and first remeasurement period. The reported *R* ^2^ is from a linear mixed effects model with plot as a random effect to account for the measurement of change in multiple microplots within the same plot.

## Data Availability

The data used in this analysis was shared with the authors by the National Forest Inventory through a data use agreement and the data can be requested from them. Our analysis code is available on Zenodo at https://doi.org/10.5281/zenodo.17976777 and Github at https://github.com/robertwbuchkowski/designing_national_forest_inventories.
