# Supplementary material for: Designing National Forest Inventories for Accurate Estimation of Soil Carbon Change
Source: Glob Chang Biol. 2026 Apr 27;32:e70868. doi: 10.1111/gcb.70868 (PMC13112338; doi:10.1111/gcb.70868)
Supplement: Supplementary file 1 — Table S1: The seven central questions of our analysis and answers to them based on analyzing Canada's National Forest Inventory Data. Figure S1:. The carbon stock across mineral and organic horizons by sampling month to demonstrate that the time of the snow‐free season in which a sample was collected did not lead to a systematic bias in the carbon measurement in the Canadian National Forest Inventory Dataset. Figure S2: The minimum detectable difference (MDD) in soil carbon stocks and rate of change using the National Forest Inventory remeasurement data set across different choices for significance level (α; left column) and statistical power (right column). The top row reports MDD using the spatial variation from the first measurement and the second one separately; the bottom row reports the MDD of change over a 10‐year period using the variation in rates of change of soil carbon stocks between the first (i.e., initial) and second (remeasurement) times. Figure 3 in the main text corresponds to α = 0.05 and power = 0.8. Figure S3:. Regression to the mean patterns for changes in carbon stock across all the National Forest Inventory plots (A). Points represent a plot mean (across microplots) for the initial carbon stock and change in total carbon stock between the initial and first remeasurement (N = 532 plots). (B) The relationship between mean carbon stock, calculated from the initial and first remeasurement data, adjusted linearly to 10‐years after the first sample. The disappearance of the negative relationship in A is suggestive of the relationship in A being a statistical artifact of sampling a spatially variable outcome, which gives rise to a pattern referred to as regression to the mean. Figure S4:. The difference in total soil carbon stock between plots with different numbers of microplots. The cyan diamonds show the mean values. Statistical significance was evaluated with a generalized linear mixed effects model with plot as a random effect to account for mu [file GCB-32-e70868-s001.docx]

Supplementary Information

Designing national forest inventories for accurate estimation of soil carbon change

Robert W. Buchkowski, Alexander Polussa, and Mark A. Bradford

Contents: One table and five additional figures.

***Table S1****: The seven central questions of our analysis and answers to them based on analyzing Canada’s National Forest Inventory Data.*

| Question | Answer | Location of Evidence |
| --- | --- | --- |
| What is the relative magnitude of between- and within-plot variation in soil properties under the NFI protocol? | They are similar for spatial stocks. Within plot variation is larger when measuring change. | Figure 2 |
| Does the minimum detectable difference (MDD) in carbon stocks and rates of change differ between mineral and organic horizons? | Organic horizon MDD is ~50% larger for spatial stocks and ~10% larger for rates of change. | Figure 3 |
| How does increasing the number of plots or microplots per plot affect the MDD? | Both reduce MDD, but increasing microplots is not as effective. | Figure 4 |
| Does grouping plots by expected covariates of soil carbon stock reduce measurement variation and MDD? | Minimal benefits because each ecozone still has quite high variability. | Figure 5 |
| Do sequential estimates of soil carbon change show a directional change? | No, the correlation between intervals is weak. | Figure 6 |
| Are soil carbon stocks biased at sites with fewer sampled microplots? | Yes for mineral horizons. Sites with fewer microplots have more soil carbon. | Figure S3 |
| Can change in percent carbon be used as a proxy for change in total carbon without collecting bulk density data? | No, the correlated change is weak. | Figure 7 |

*
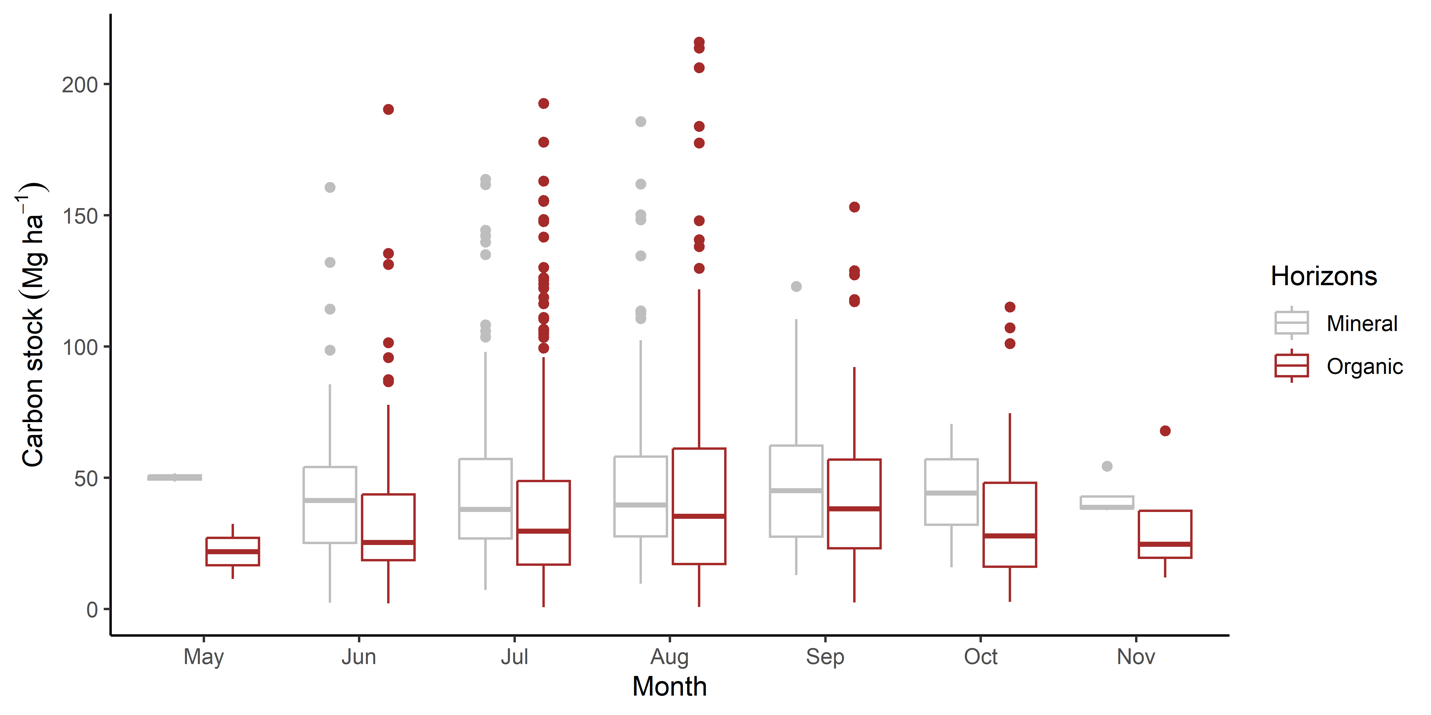
*

***Figure S1****: The carbon stock across mineral and organic horizons by sampling month to demonstrate that the time of the snow-free season in which a sample was collected did not lead to a systematic bias in the carbon measurement in the Canadian National Forest Inventory Dataset.*


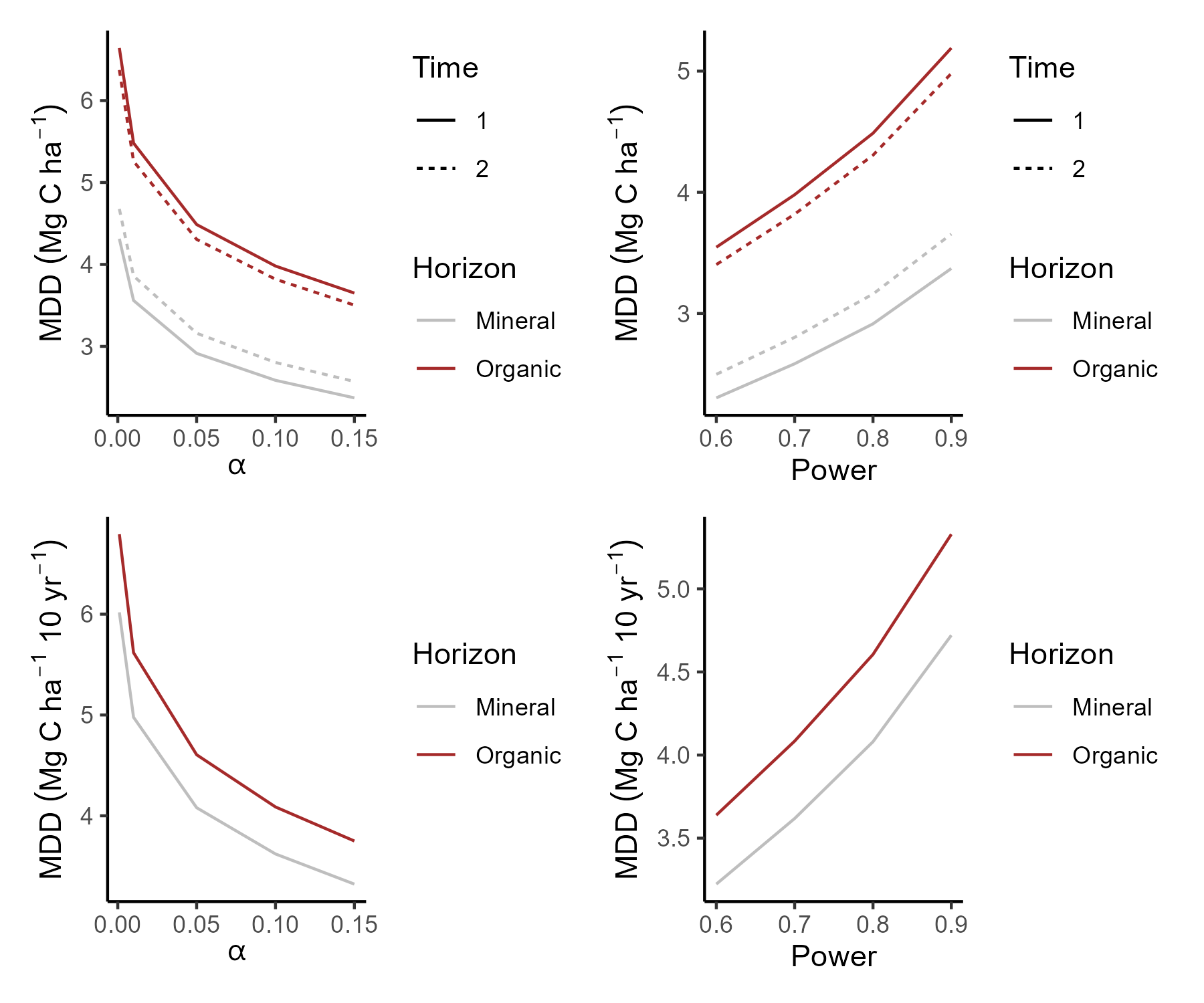


***Figure S2:*** *The minimum detectable difference (MDD) in soil carbon stocks and rate of change using the National Forest Inventory remeasurement data set across different choices for significance level (*$\alpha$*; left column) and statistical power (right column). The top row reports MDD using the spatial variation from the first measurement and the second one separately; the bottom row reports the MDD of change over a 10-year period using the variation in rates of change of soil carbon stocks between the first (i.e. initial) and second (remeasurement) times. Figure 3 in the main text corresponds to* $\alpha$*= 0.05 and power = 0.8.*


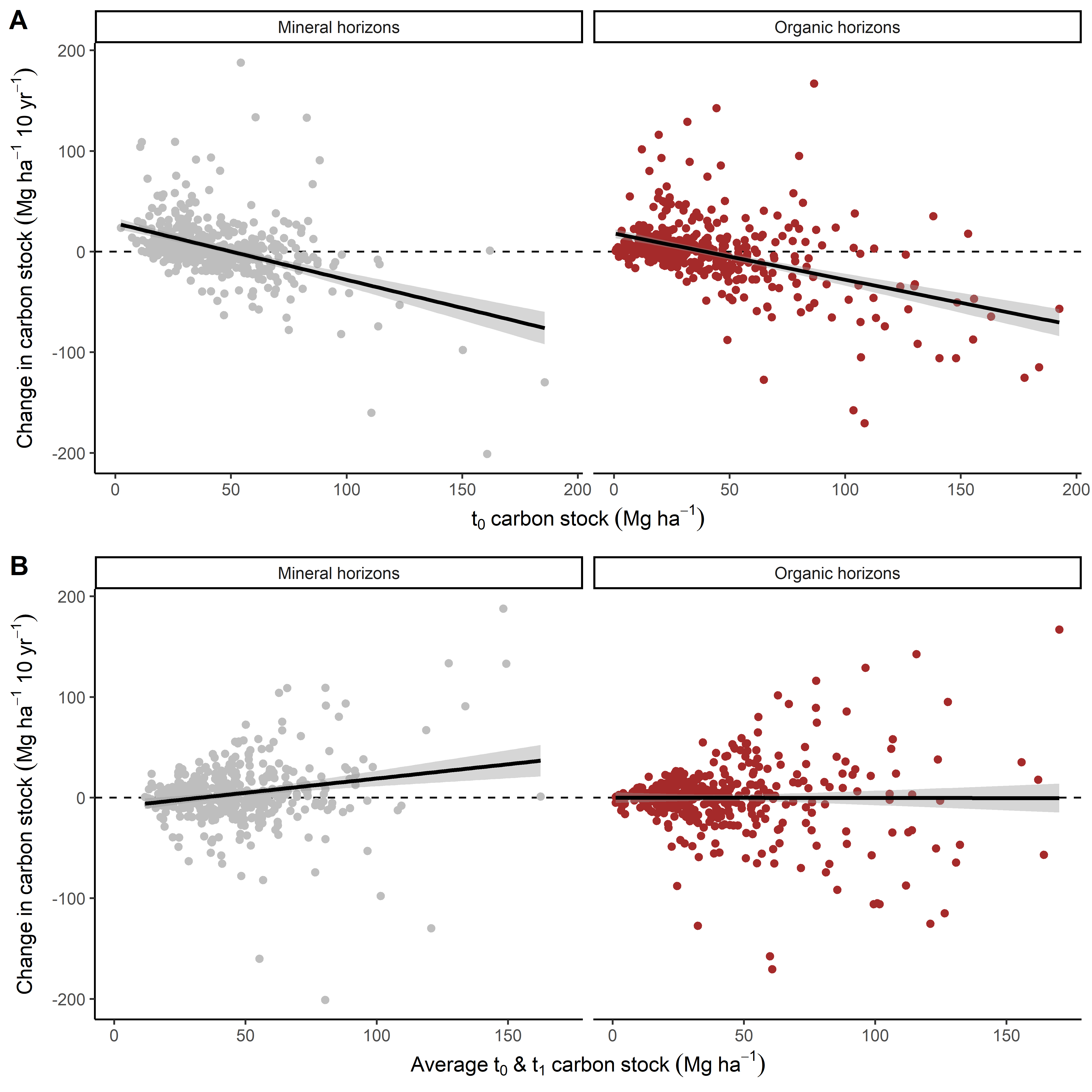


***Figure S3****: Regression to the mean patterns for changes in carbon stock across all the National Forest Inventory plots (A). Points represent a plot mean (across microplots) for the initial carbon stock and change in total carbon stock between the initial and first remeasurement (N=532 plots). (B) The relationship between mean carbon stock, calculated from the initial and first remeasurement data, adjusted linearly to 10-years after the first sample. The disappearance of the negative relationship in A is suggestive of the relationship in A being a statistical artifact of sampling a spatially variable outcome, which gives rise to a pattern referred to as regression to the mean.*


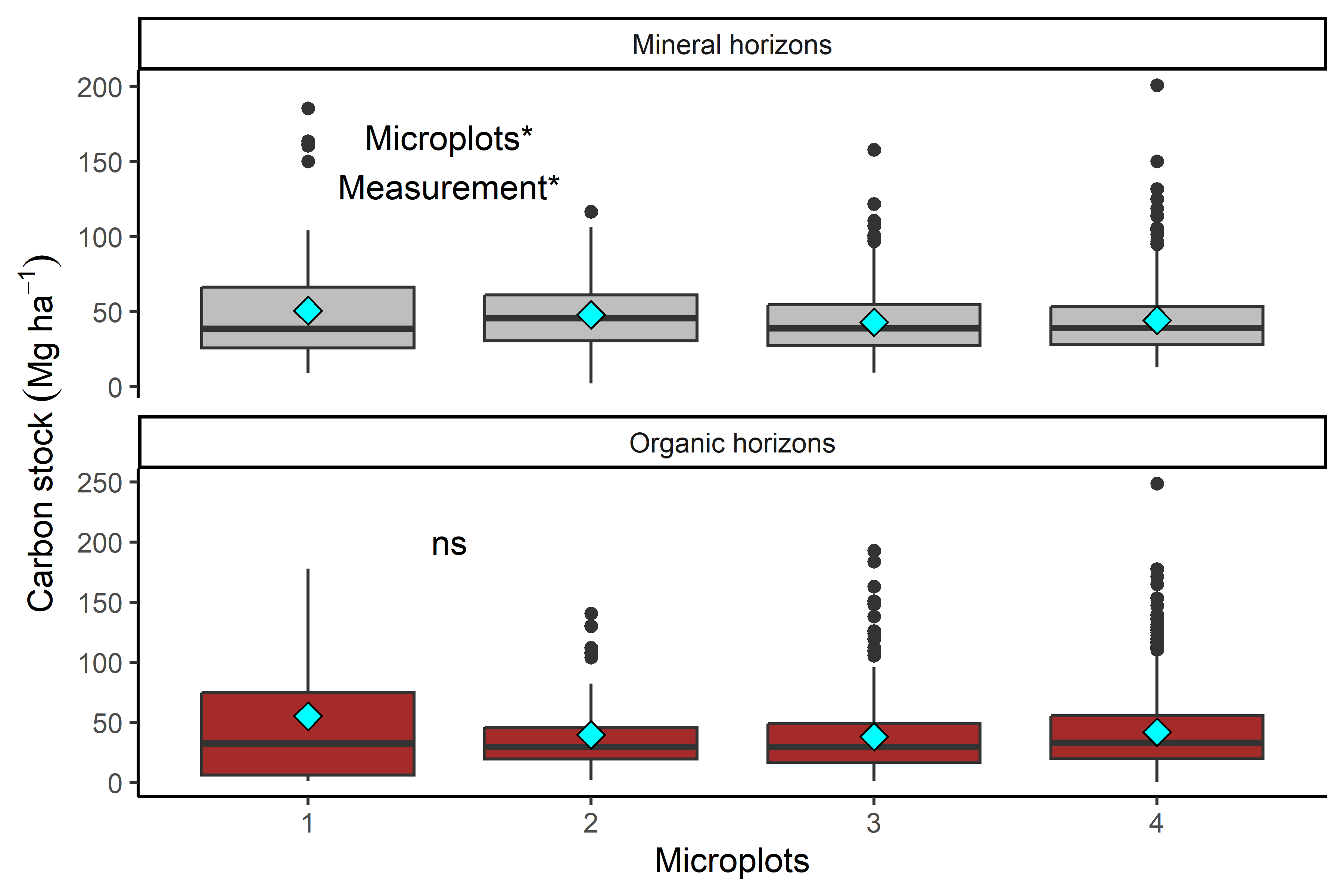


***Figure S4****: The difference in total soil carbon stock between plots with different numbers of microplots. The cyan diamonds show the mean values. Statistical significance was evaluated with a generalized linear mixed effects model with plot as a random effect to account for multiple measurements per plot. For the mineral horizon, mean total carbon decreased as the number of microplots increased with plots with one microplot having 5.6 Mg ha^-1^ more carbon than the average plot.*


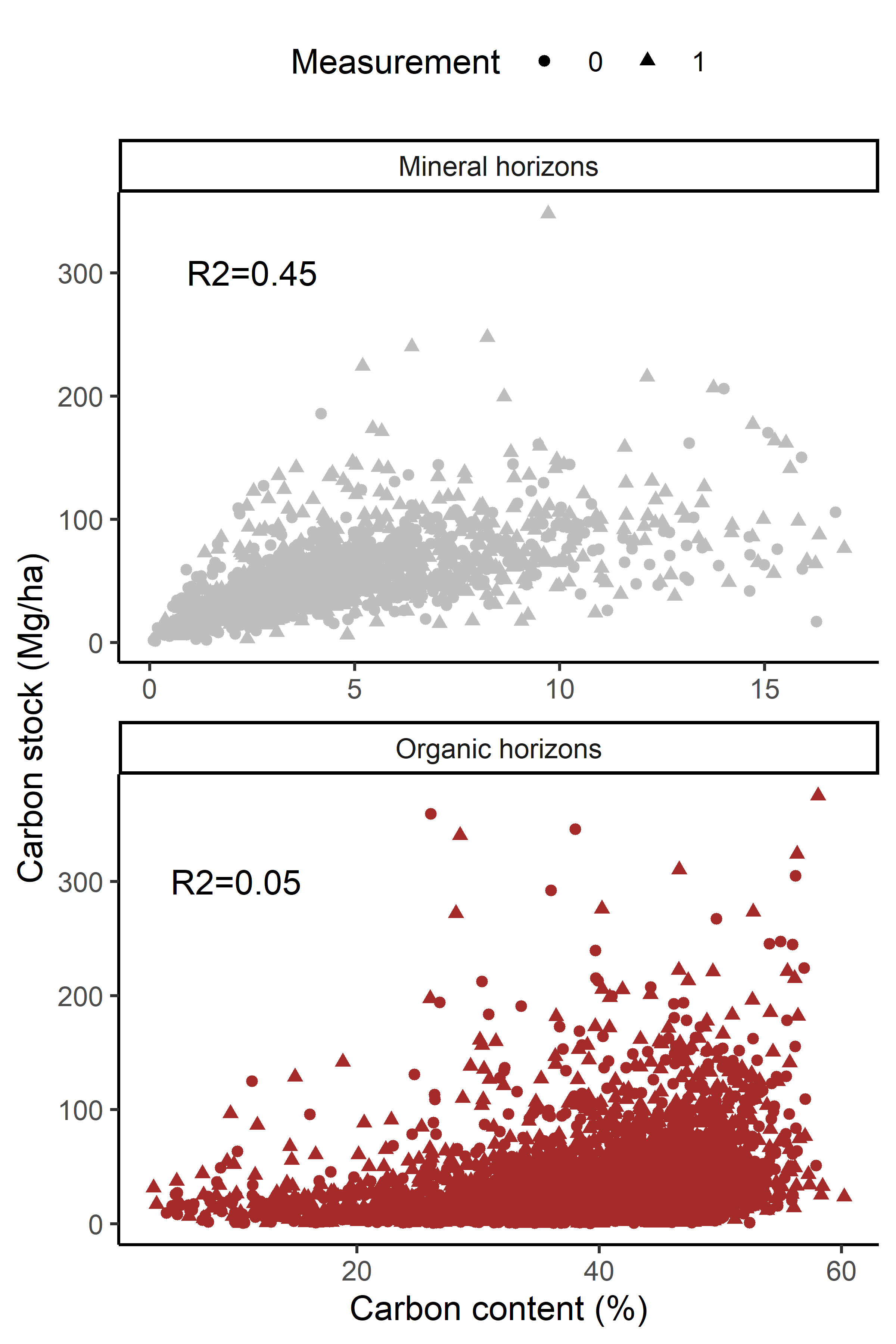


***Figure S5****: The relationship between carbon concentrations and stocks for the initial measurement and first remeasurement period. The reported R2 is from a linear mixed effects model with plot as a random effect to account for the measurement of change in multiple microplots within the same plot.*
